# Study on the effect of different high-voltage electric field polarization process parameters on the vitality of dried chili pepper seeds

**DOI:** 10.1038/s41598-024-57978-z

**Published:** 2024-03-27

**Authors:** Sheng Sun, Bin Hu, Xinming Wu, Xin Luo, Mengyu Guo, Hanjun Liu

**Affiliations:** 1 College of Mechanical and Electronic Engineering, Shihezi University, Shihezi, 832003 China; 2 Xinjiang Production and Construction Corps Key Laboratory of Modern Agricultural Machinery, Shihezi, 832003 China; 3 Key Laboratory of Northwest Agricultural Equipment, Ministry of Agriculture and Rural Affair, Shihezi, 832003 China

**Keywords:** High voltage electric field, Polarisation process, Seed viability, Conductivity, Germination test, Plant breeding, Plant ecology

## Abstract

To study the effect of different high-voltage electric field polarisation treatment process parameters on the viability of seeds of dried chili peppers. In this study, a high-voltage electrostatic polarisation treatment system was constructed to carry out experiments on the effects of different high-voltage electric field polarisation treatment process parameters on the viability of dried chili seeds. Conduct one-way tests to determine the preferred polarisation method and the preferred interval for output voltage and polarisation time. Two-factor, five-level central combination test with output voltage and polarization time as test factors and seed conductivity as a response indicator. Determining the better combination of parameters for output voltage and polarization time; Conducting seed germination trials to validate the effectiveness of the polarisation process. The results of the one-way test showed that: Negative-voltage polarisation was more effective than positive-voltage polarisation and alternating positive–negative-voltage polarisation in promoting seed vigor, with a better output voltage in the range of 10–14 kV, and a better polarisation time in the range of 20–40 s; The results of orthogonal tests showed that: Under the condition of negative voltage polarisation treatment, the output voltage of 12.08 kV and polarisation time of 30.32 s was the better parameter combination, at which the seed conductivity was minimum 159.87 uS/(cm g). Analyzing the function of cell membrane selective semi-permeability by seed conductivity change and revealing the mechanism of seed viability enhancement by high voltage electric field polarisation treatment; In the seed germination test, compared with the control group, seed germination potential increased by 9.09%, germination rate increased by 20.45%, germination index increased by 3.49, and vigor index increased by 41.66 under high-voltage electrostatic polarisation treatment, and all vigor indexes were significantly improved. The results of this study can provide a basis for the selection of processes and parameters for subsequent high-voltage electric field polarisation treatment of crop seeds.

## Introduction

Because of the long hours of sunshine and the large temperature difference between day and night, Xinjiang grows dried chilies with high yield, good quality, and high content of dry matter and red pigment. It is an important production area and processing base for dried chilies in China^1,2^. According to the 2022 Xinjiang Yearbook statistics 2021 mechanized planting area of 600,000 mu, with an annual output of more than 250,000 tonnes of dried peppercorns, the annual output of dried peppercorns accounted for 1/5 of the nation's.

The mechanism of germination and growth of crop seeds is an objective law of nature. It is highly susceptible to seed conditions and environmental disturbances that can have a significant impact on later crop growth and development^3–5^. High-quality seeds are the basis for crop quality and high yields^6–9^. Consequently. To regulate seed germination and its growth and development process, crop seed treatment methods have become a hot topic of research. Among them, the high-voltage electrostatic field treatment of seeds based on electromagnetobiology has triggered much research. Relevant studies have shown that: Reasonable electric field strength and polarisation treatment time can improve crop germination and seedling quality^10–13^. Altering endogenous hormone levels during seed germination^14,15^. Altering the permeability of cell membranes^16–19^. Mitigating the inhibitory effect of salt stress on crop growth^20,21^. Increase seed enzyme activity^22–24^. Improvement of seed resistance^25,26^. Altered seed endophytic bacterial communities^27^. Thereby increasing seed viability. In addition, crop seeds treated with electric fields can improve their disease resistance and yield^28,29^. All of the above researchers have achieved some results in this field. However, little has been reported on the effect of different high-voltage electric field polarisation process parameters on the viability of dried chili seeds based on different polarisation methods.

In this study, we constructed a high-voltage electrostatic polarisation treatment system. Effect of carrying out different process parameters of high voltage electric field polarisation treatment on the viability of seeds of dried chili pepper production. Single-factor test with high-voltage electrostatic polarisation mode, high-voltage electrostatic generator output voltage, and high-voltage electric field polarisation time as test factors respectively. Determine the preferred polarisation method and the preferred interval for output voltage and polarisation time; Based on a superior high-voltage electrostatic polarisation method. A two-factor, five-level central combination test was conducted using the output voltage of the high-voltage electrostatic generator the polarization time of the high-voltage electric field as the test factors, and the seed conductivity as the response indicator. Determining the better combination of parameters for output voltage and polarization time; Analysis of the recovery of cell membrane selective semi-permeability function under conditions of high-voltage electric field polarisation treatment by seed conductivity changes. Uncovering the mechanism of high-voltage electric field polarisation treatment to enhance the viability of dried chili seed production; Conducting seed germination tests. Verification of seed viability under conditions of high-voltage electric field polarisation treatment. The results of this study can provide a basis for the selection of processes and parameters for subsequent high-voltage electric field polarisation treatment of crop seeds. It may also serve as a reference for more scholars to study the effects of electric fields on the biological effects of crop seeds.

## Materials and methods

### Test materials and test environment

The dried chili seed used in the experiment was King Nongkang Dry Chili. It has a varietal purity of not less than 95.0%, a varietal clarity of not less than 99.0%, and a moisture content of not more than 13%. Its implementation standard is GB16715.3–2010. It was produced by Xinjiang Nongren Seed Science and Technology Limited Liability Company in 2015, with a seed quality assurance period of 12 months, and originated from No. 333 Wuyidong Road, Changji City, Xinjiang. It is distributed by Xinjiang Farmers Shihezi Branch General Distribution. High-voltage electric field polarisation treatment for making dried chili seeds was tested in the precision sowing laboratory of Shihezi University. The germination test of prepared dried chili seeds was carried out in the analytical and testing laboratory of Shihezi University. The start date for the High Voltage Electric Field Polarisation (HVEFP) treatment of the Dried Chili Seed Preparation (DCP) test and the DCP seed germination test was September 2023.

### Test equipment

#### High voltage electrostatic polarisation system

**Figure 1:**
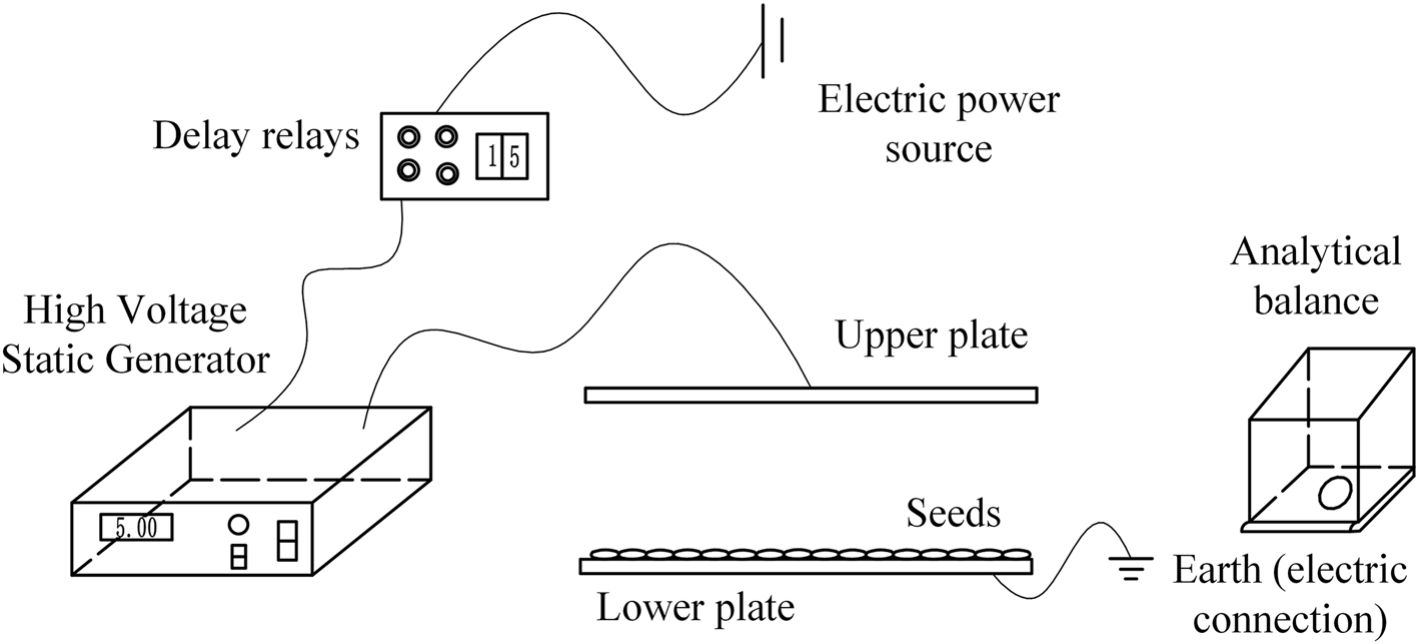
Schematic diagram of high voltage electrostatic polarisation treatment system.

The high-voltage electrostatic polarisation treatment system mainly consists of a high-voltage electrostatic generator, a pole plate, a time-delay relay, and an analytical balance. One of the high-voltage electrostatic generator output voltages can be continuously and infinitely adjustable, the output pulse peak voltage of 0–120 kV, which is manufactured by Shenzhen Chuanqi machinery enterprises; The time-delay relay is DH48S-1Z, it has a time-delay range of 0.01 s-99 h, it has a control accuracy of ≤ 0.2% ± 0.05 s, and it is marketed by International Industrial Automation Enterprises; The material of the pole plate is stainless steel, the length of the pole plate is 200 mm, the width is 200 mm and the thickness is 1 mm. The distance between the upper and lower plates is 30 mm; The analytical balance is a BSM-120.4, which has an accuracy of 0.0001 g, a repeatability error of ± 0.0001 g, and a linearity of ± 0.0002 g. The analytical balance is a BSM-120.4. As shown in Fig. 1.

When at work. Dried chili seeds were placed flat on the lower pole plate. Adjust the delay relay to the polarization time. Turn on the high-voltage electrostatic generator polarisation treatment to make dried chili seeds. Remove the seeds at the end of polarisation. Determination of its initial conductivity. Carrying out the next polarisation process.

#### Conductivity tester

The conductivity tester used in the test was a DDS-11A conductivity meter with a range of 0.00 μS/cm—199.9 μS/cm, and the basic error of the electronic unit was ± 1.0% (FS), and the basic error of the instrument was ± 2.0% (FS).

#### Artificial climatic chamber

The artificial climate chamber used in the test was RGX-300E, produced by Tianjin Taiste Instrument Co. Its temperature control range is: 0–65℃ when there is no light, 10–65℃ when there is light, temperature fluctuation ± 1℃; humidity control range is 40–90% RH, humidity deviation is ± 5–7%; illuminance is 0–15,000 LX, and working temperature is 5–35℃.

### Test methods

#### Seed viability evaluation methods

The method of determining seed viability in the experiment was the conductivity method recommended by the Committee on Vigor Determination of the International Association of Seed Testing (IAST) in 1995. The principle is that the seed absorbs swelling and fission before germination, resulting in changes in the structure of the cell membrane, denaturation of lipoproteins, molecular rearrangement, and increased permeability coefficients. Increased extravasation of electrolytes such as amino acids and organic acids within it. Under the action of the electric field, the charged ions in the exudate move directionally, transferring electrons with a conductive effect^30^. Typically, the smaller the electrolyte content of the water, the lower the conductivity and the higher the seed viability.

When seed conductivity is measured. Analytical balance for weighing dried chili seeds. Assuming a mass of m (accuracy 0.0001 g); Rinse the seeds three times in pure water. Remove surface water with filter paper. Into a beaker. Add 50 mL of purified water. Determination of its initial conductivity *σ*_*1*_, μS/(cm·g); After 24 h of resting. Determination of seed leachate conductivity *σ*_*2*_, μS/(cm·g). Seed conductivity is assumed to be σ, μS/(cm·g). Then there is

1 $$\sigma = \frac{{\sigma_{2} - \sigma_{1} }}{m}$$

#### One-way experiment

Three groups of dried chili seeds were randomly selected, 1000 seeds per group. The analytical balance was weighed in turn and the masses were 5.7242 g, 5.6739 g, and 5.7018 g. Find the mean value of 5.7000 g. During the experiment, to facilitate the calculation of seed conductivity. 5.7000 g of dried chili seeds were taken from each group of high voltage electric field polarisation treated dried chili seed test group and control group.

Several dried chili seeds were randomly selected and the mass of seeds in each group was 5.7000 g. To determine the preferred high voltage electrostatic polarisation method the preferred interval for the output voltage of the high voltage electrostatic generator and the polarization time of the high voltage electric field. The following three major groups of one-way tests were conducted separately: 1) Investigating the effect of different high-voltage electrostatic polarisation on the viability of dried chili seed production. The trial was divided into 3 groups. At a high voltage electrostatic generator output voltage of 10 kV and a high voltage electric field polarisation time of 15 s. Dried chili seeds were treated sequentially with positive output voltage polarisation (15 s), negative output voltage polarisation (15 s), and alternating positive–negative output voltage polarisation (positive output voltage polarisation for 7.5 s followed by negative output voltage polarisation for 7.5 s). Determination of its conductivity. Each group of tests was repeated three times, and the average value was taken as the result of that group of tests; 2) Explore the effect of different output voltages of high voltage electrostatic generator on the viability of dried chili seeds. The trial was divided into 10 groups. Under negative output voltage polarisation and high voltage electric field polarisation time of 15 s. Dried chili seeds were treated with output voltages of 2 kV, 4 kV, 6 kV, 8 kV, 10 kV, 12 kV, 14 kV, 16 kV, 18 kV and 20 kV in that order. Determination of its conductivity. Each group of tests was repeated three times, and the average value was taken as the result of that group of tests; 3) Investigating the effect of different high voltage electric field polarisation times on the viability of dried chili pepper seed production. The trial was divided into 9 groups. Under negative output voltage polarisation and high voltage electrostatic generator output voltage of 10 kV. Dried chili seeds were treated sequentially with polarisation times of 10 s, 20 s, 30 s, 40 s, 50 s, 60 s, 70 s, 80 s and 90 s. Determination of its conductivity. Each group of tests was repeated three times, and the average value was taken as the result of that group of tests.

#### Centre combination design experiment (CCDE)

Based on the results of the one-way test. Negative output voltage polarisation outperforms positive output voltage polarisation and alternating positive–negative output voltage polarisation in enhancing the viability of dried chili seeds. The better interval of the output voltage of a high-voltage electrostatic generator for enhancing the viability of dried chili seed preparation was 10–14 kV. The preferred interval of high voltage electric field polarisation time for enhancing the viability of prepared dried chili seeds was 20–40 s. Consequently. Under conditions of negative output voltage polarization. A central combination of high-voltage electrostatic generator output voltage and high-voltage electric field polarisation time as test factors and dried chili seed conductivity as a response indicator was used for the test. The coded values of the test factors are detailed in Table 1. By the design requirements of the Central Composite trial, the trial was divided into 13 groups. Each group of tests was repeated three times, and the average value was taken as the result of that group of tests. Data were processed using Design Expert 10.0.4 software. Establishment of fitted regression equations between each test factor and the response indicator. Determination of optimal combinations of high-voltage electrostatic generator output voltage and high-voltage electric field polarisation time parameters. Provide a basis for the selection of process parameters for high-voltage electric field polarisation treatment systems.

**Table 1:** Experimental factor coding table.

Coded Value	Considerations	
	Output voltage X_1_/kV	Polarisation time X_2_/s
− 1.41421	9.17157	15.8579
− 1	10	20
0	12	30
1	14	40
1.41421	14.8284	44.1421

#### Dried chili seed germination test

To verify the effect of high-voltage electric field polarisation treatment in enhancing the viability of dried chili seeds. Several dried chili seeds were randomly selected and the mass of seeds in each group was 5.7000 g. Conducting germination tests of dried chili seeds. The experiment was divided into two groups as follows: 1) Based on the optimization results of the experimental parameters of the central combination design. Under the condition of negative output voltage polarization, the preferred combination of process parameters of the high voltage electric field polarisation treatment system is a high voltage electrostatic generator output voltage of 12.08 kV and high voltage electric field polarisation time of 30.32 s. To control the high voltage electric field polarisation time, the high voltage electric field polarisation time is rounded off to 30 s. Dried chili seeds were prepared by polarisation treatment at this preferred parameter; 2) No high-voltage electric field polarisation treatment, was considered as a control group. Each set of tests was repeated 3 times. The average value of each seed viability evaluation index was taken as the result of the experiment.

High-voltage electric field polarisation-treated seeds and controls were planted separately in cavity trays. Burrow trays were placed in an artificial climatic chamber for seed germination tests. The parameters of the simulated germination environment in the experiment were based on national standards: Constant temperature of 25 °C, continuous light, and 100% relative humidity (GB/T 3543.4–1995). Dried chili seeds should be rehydrated regularly during germination. Adequate water for seed germination. Timely cleaning and removal of moldy seeds during germination tests. So as not to interfere with the growth of other seedlings. The number of germination of each group of specimens was counted from the 3rd day of the germination test. Seedlings were washed on day 14 of the germination test. Measurement data according to seed testing standards^25^. Measurement of all seedlings. The viability indexes of prepared dried chili seeds including germination percentage, germination potential, germination index, and vigor index were calculated for each group of specimens.

2 $$\begin{gathered} GE = \frac{{G_{4} }}{N} \hfill \\ GP = \frac{{G_{14} }}{N} \hfill \\ GI = \sum \frac{{G_{t} }}{{D_{t} }} \hfill \\ VI = GI\cdot{\text{S}} \hfill \\ \end{gathered}$$

Which: *GE* is germination potential, %; *GP* is germination rate, %; *GI* is the germination index; *VI* is vigor index, cm; *L* is the length of the seedling, cm; *D*_*t*_ is the number of days in the germination test; *G*_*4*_ is the number of germination of manufactured dried chili seeds at the time of counting on day 4; *G*_*14*_ is the number of normal seedlings of dried chili seeds at the time of washing and counting on day 14; *G*_*t*_ is the number of germination at the time of counting the days to germination corresponding to *D*_*t*_; *N* is the number of dried chili seeds in the germination test; *S* is the length of the seedling, cm.

#### Seedling indicator measurements and data processing methods

Remove the seedlings from the cavity trays and clean them using pure water. Remove water from the surface of seedlings with absorbent paper. Measurement of the length of cleaned seedlings with a digital vernier caliper (Model Mitutoyo 500–172-30, measuring range 0–200 mm, accuracy ± 0.001 mm). The average length of all seedlings was taken as the result of the test group. Comparison with the control group. Validation of high-voltage electrostatic fields for the promotion of seed viability in dried chili production.

### Research statements involving plants

The dried chili seed used in the experiment was King Nongkang Dry Chili. It has a varietal purity of not less than 95.0%, a varietal clarity of not less than 99.0%, and a moisture content of not more than 13%. Its implementation standard is GB16715.3-2010. It was produced by Xinjiang Nongren Seed Science and Technology Limited Liability Company in 2015, with a seed quality assurance period of 12 months, and originated from No. 333 Wuyidong Road, Changji City, Xinjiang. It is distributed by Xinjiang Farmers Shihezi Branch General Distribution; The method of determining seed viability in the experiment was the conductivity method recommended by the Committee on Vigor Determination of the International Association of Seed Testing (IAST) in 1995; The parameters of the simulated germination environment in the experiment were based on national standards: Constant temperature of 25 °C, continuous light and 100% relative humidity (GB/T 3543.4-1995); Seed viability indicators for each group of dried chili seeds in the experiment were measured and calculated by seed testing standards, and the corresponding data were obtained; We comply with the IUCN Policy Statement on Research Involving Species at Risk of Extinction and the Convention on the Trade in Endangered Species of Wild Fauna and Flora.

## Results and analyses

### One-way test

#### Effect of different high-voltage electrostatic polarisation methods on the viability of seeds of dried chili peppers

Different high-voltage electrostatic polarisation modes promote the viability of dried chili pepper seed production. However, there are slight differences in the effect of vigor enhancement between different polarisation styles. At a high voltage electrostatic generator output voltage of 10 kV and a high voltage electric field polarisation time of 15 s. The seed conductivity after polarisation with forward output voltage was 223.05 uS/(cm·g). The seed conductivity after polarisation with negative output voltage was 206.58 uS/(cm·g). Seed conductivity after alternating positive–negative output voltage polarisation was 232.98 uS/(cm·g). The seed conductivity of the control group was 243.94 uS/(cm·g). Seed viability after negative output voltage polarisation was higher than after positive output voltage polarisation. Seed viability after positive output voltage polarisation was higher than after alternating positive–negative output voltage polarisation. Seed viability was higher than the control after different polarisation modes. Trace something to its source: Plant cell membranes have a high positive charge on the outside of the membrane and positive and negative charges are attracted to each other, so seed cell membranes are more sensitive to negative output voltage polarisation treatments. The selective semi-permeable function of the cell membrane is restored more rapidly when stimulated by an external negative high-voltage electric field, and the corresponding extravasation of mineral ions and organic matter from the seeds during soaking is reduced. Reduced content of electrically conductive substances in seed leachate, lower seed conductivity, higher seed viability.

#### Effect of different output voltages of high-voltage electrostatic generators on the viability of seeds of dried chili pepper production

**Figure 2:**
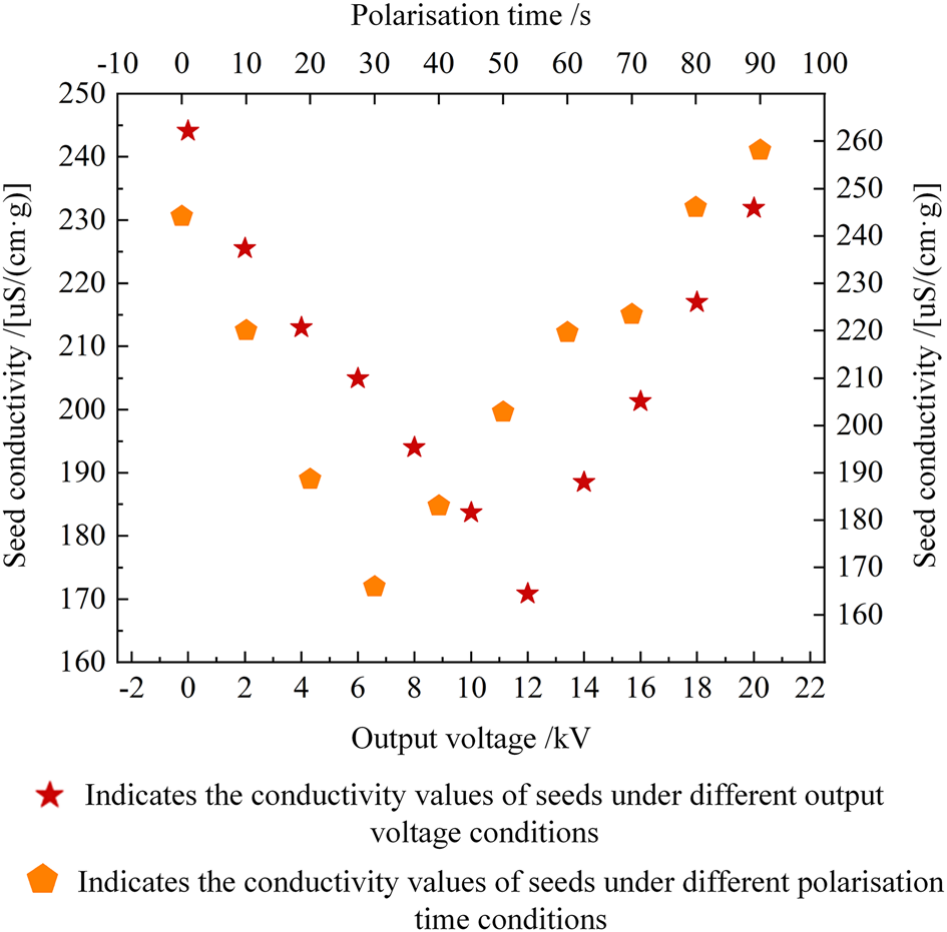
Results of one-factor test on the effect of different output voltages and polarisation times on seed viability.

The reasonable output voltage of a high-voltage electrostatic generator promotes the vigor of dried chili seeds. Under negative output voltage polarisation and high voltage electric field polarisation time of 15 s. As the output voltage of the high-voltage electrostatic generator increases. The conductivity of prepared dried chili seeds showed a trend of decreasing and then increasing changes. Seed viability showed a trend of increasing and then decreasing; When the output voltage was increased from 0 to 12 kV, the seed conductivity gradually decreased and the seed viability gradually increased; When the output voltage was increased from 12 to 20 kV, the seed conductivity gradually increased and the seed viability gradually decreased; Seed conductivity was minimized at 170.86 uS/(cm·g) when the output voltage was 12 kV, when seed viability was highest. As shown in Fig. 2; When the output voltage is 0–12 kV. High-voltage electric fields are less intense. At this point, the electric field is not sufficient to stimulate the cell membrane to fully restore its selective semi-permeable function. Leads to partial extravasation of mineral ions and organic matter from the seeds during soaking. Higher seed conductivity and lower seed viability; When the output voltage is 12–20 kV. Higher intensity of high voltage electric field. Formation of reversible perforations on the surface of the cell membrane. Leads to partial extravasation of mineral ions and organic matter from the seeds during soaking. Higher seed conductivity and lower seed viability; When the output voltage is 12 kV. Stimulation of cell membranes by electric fields maximizes the restoration of selective semi-permeable functions. Resulting in less mineral ion and organic matter extravasation from the seeds during soaking. Lower seed conductivity and higher seed viability.

#### Effect of different high voltage electric field polarisation times on the viability of dried chili pepper seed production

Reasonable polarisation time of high voltage electric field promotes the viability of dried chili seed production. However, prolonged polarisation can inhibit seed viability^30^. Under negative output voltage polarisation and high voltage electrostatic generator output voltage of 10 kV. As the polarisation time of the high-voltage electric field increases. The conductivity of prepared dried chili seeds showed a trend of decreasing and then increasing changes. Seed viability showed a trend of increasing and then decreasing; As the polarisation time increased from 0 to 30 s, seed conductivity gradually decreased and seed viability gradually increased; When the polarisation time was increased from 30 to 90 s, the seed conductivity gradually increased and the seed viability gradually decreased; The minimum seed conductivity was 165.96 uS/(cm·g) when the polarisation time was 30 s, when the seed viability was the highest. As shown in Fig. 2; High-voltage electric field polarisation time is shorter when the polarisation time is 0–30 s. At this point, the electric field is not sufficient to stimulate the cell membrane to fully restore its selective semi-permeable function. Resulting in partial extravasation of mineral ions and organic matter from the seeds during soaking, higher seed conductivity, and lower seed vigor. When the polarisation time is 30–90 s, the high-voltage electric field polarisation time is longer. Formation of reversible perforations on the surface of the cell membrane. Resulting in partial extravasation of mineral ions and organic matter from the seeds during soaking, higher seed conductivity, and lower seed vigor; When the polarisation time of the high voltage field is 30 s. Stimulation of cell membranes by electric fields maximizes the restoration of selective semi-permeable functions. Resulting in less mineral ion and organic matter extravasation from the seed during soaking, lower seed conductivity, and higher seed vigor.

### Centre combination test

#### Results and analysis of the central combination test

The central combination test protocol and results are shown in Table 2 and the ANOVA is shown in Table 3. Data were processed using Design Expert 10.0.4 software. The fitted regression equation between seed conductivity and high voltage electrostatic generator output voltage X_1_ and high voltage electric field polarisation time X_2_ was established as:

3 $$Y = 951.253 - 93.142X_{1} - 15.083X_{2} + 0.578X_{1} X_{2} + 3.129X_{1}^{2} + 0.134X_{2}^{2}$$

**Table 2:** Orthogonal test scheme and results.

Run	Output voltage X_1_/kV	Polarisation time X_2_/s	Seed conductivity Y/[uS/(cm g)]
1	10	20	198.21
2	9.17157	30	189.66
3	12	44.1421	185.25
4	12	30	158.22
5	12	30	166.19
6	12	30	155.58
7	12	30	157.81
8	12	30	161.79
9	14	40	195.84
10	10	40	172.17
11	12	15.8579	188.66
12	14	20	175.64
13	14.8284	30	180.89

**Table 3:** Analysis of variance of orthogonal experiment results.

Source	Seed conductivity			
	Sum of Squares	df	F-value	P-value
Model	2627.11	5	39.65	< 0.0001
X_1_	15.97	1	1.21	0.3086
X_2_	14.21	1	1.07	0.3348
X_1_X_2_	534.53	1	40.34	0.0004
X_1_^2^	1089.74	1	82.24	< 0.0001
X_2_^2^	1240.92	1	93.65	< 0.0001
Residual	92.75	7		
Lack of fit	23.76	3	0.4593	0.7256
Pure error	68.99	4		
Cor total	2719.86	12		

Significance analysis of regression models. From the ANOVA table, it can be seen that the model of seed conductivity, an indicator of experimental evaluation, was highly significant (P < 0.01), the misfit term was not significant (P > 0.05), and the regression equation was not misfit. Removing the insignificant terms from the regression model, the fitted regression equation is obtained as:

4 $$Y = 951.253 + 0.578X_{1} X_{2} + 3.129X_{1}^{2} + 0.134X_{2}^{2}$$

At this point, the regression model fit coefficient of determination R_2_ is 0.9659 respectively. Indicates a high correlation between regression model predictions and actual values. The p-value of the misfit term was 0.7256 greater than 0.05 respectively, indicating that the regression equation was well-fitted. This equation can be used to optimize the process parameters of a high-voltage electric field polarisation treatment system.

**Figure 3:**
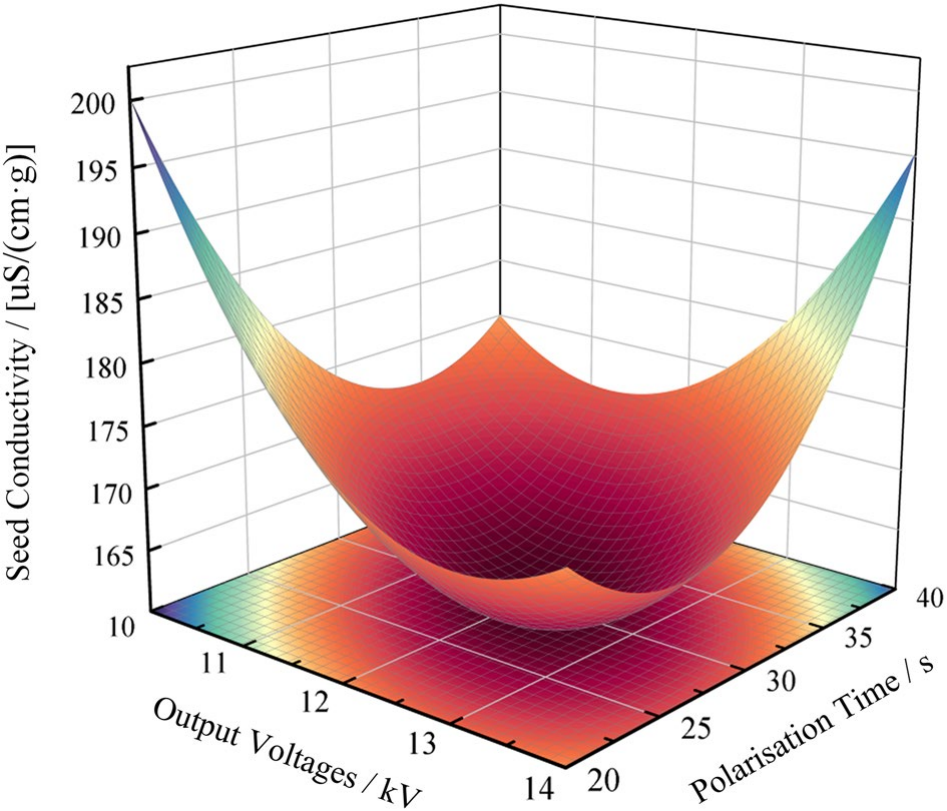
Response surface plot of the effect of the interaction term on seed conductivity.

Based on the analysis of variance (ANOVA) of the test results in Table 3, it was shown that: The primary and secondary order of effect of test factors on seed conductivity was output voltage and polarisation time. The interaction term X_1_X_2_ of output voltage and polarization time had a significant effect on seed conductivity. As shown in Fig. 3.

Based on Fig. 3. When the output voltage of the high-voltage electrostatic generator is 12 kV the time of the high-voltage electric field polarisation treatment is gradually increased. Seed conductivity showed a trend of decreasing and then increasing, and seed viability showed a trend of increasing and then decreasing. At an increase in the time of the high voltage electric field polarisation treatment from 20 s to 30.504 s. Gradual decrease in seed conductivity and gradual increase in seed viability; At an increase in the time of polarisation treatment in the high voltage electric field from 30.504 s to 40 s. Gradual increase in seed conductivity and gradual decrease in seed viability. Seed conductivity had a minimum value of 159.885 uS/(cm·g) at a high voltage electric field polarisation treatment time of 30.504 s, when seed viability was highest; When the high-voltage electric field polarisation treatment time is 30 s and the output voltage of the high-voltage electrostatic generator gradually increases. Seed conductivity showed a trend of decreasing and then increasing, and seed viability showed a trend of increasing and then decreasing. When the output voltage of the high-voltage electrostatic generator is increased from 10 to 12.114 kV. Gradual decrease in seed conductivity and gradual increase in seed viability; When the output voltage of the high-voltage electrostatic generator is increased from 12.114 kV by 14 kV. Gradual increase in seed conductivity and gradual decrease in seed viability. Seed conductivity had a minimum value of 159.878 uS/(cm·g) at a high voltage electrostatic generator output voltage of 12.114 kV when seed viability was highest.

#### Optimisation of polarisation process parameters

To obtain an optimal combination of process parameters for the high-voltage electrostatic polarisation treatment system. Minimum conductivity as an objective function, when seed viability is highest. Combining boundary conditions for each test factor. Objective-optimized design of regression models for seed viability evaluation indicators. The optimization objective function and constraints are:

5 $$\left\{ \begin{gathered} 10\begin{array}{*{20}c} {} \\ \end{array} kV \le X_{1} \le 14\begin{array}{*{20}c} {} \\ \end{array} kV \hfill \\ 20\begin{array}{*{20}c} {} \\ \end{array} s \le X_{2} \le 40\begin{array}{*{20}c} {} \\ \end{array} s \hfill \\ minimize\begin{array}{*{20}c} {} \\ \end{array} Y(X_{1} ,X_{2} ) \hfill \\ \end{gathered} \right.$$

Substituting the objective function and constraints into Design-Expert 10.0.4 yields the optimal combination of parameters for the test factors: high voltage electrostatic generator output voltage of 12.08 kV and high voltage electric field polarisation time of 30.32 s. At this time, the seed conductivity is minimized to 159.87 uS/(cm·g), and the seed viability is the highest.

### Dried chili seed germination test

The number of germination of the seeds of dried chilies was counted on the 4th day and the number of seeds germinated was 11 in the high voltage electric field polarisation treated test group and 3 in the non-high voltage electric field treated control group; The number of germination of the seeds of dried chilies was counted at the 14th day and the number of seeds germinated was 74 in the experimental group treated with high voltage electric field polarisation and 56 in the control group not treated with high voltage electric field; The length of the dried chili seedlings was measured by washing the seedlings at day 14, I in Table 4 is the length of the chili seedlings of the HVF polarisation treated test group and II in Table 4 is the length of the chili seedlings of the untreated HVF control group.

**Table 4:** Length of dried chili seedlings.

I/mm	74.85	76.94	75.74	78.65	59.16	76.76	80.04	91.97	89.11	86.02	89.05	86.12	76.32
	73.73	58.5	88.94	89.25	82.65	78.75	86.35	86.32	94.76	96.36	98.05	89.75	76.89
	93.66	85.49	80.55	103.41	88.93	77.23	82.54	87.36	75.71	65.83	82.41	86.22	88.33
	87.98	78.7	109.75	79.45	90.23	84.19	89.45	76.89	86.32	78.89	86.75	75.53	92.54
	81.31	75.81	86.43	79.65	94.99	85.87	102.55	81.96	88.45	89.91	77.36	86.54	79.75
	83.74	83.75	84.21	84.32	88.75	75.74	85.75	95.56	85.98				
II/mm	84.15	57.87	61.17	72.78	63.45	67.54	63.64	60.52	61.59	65.72	57.78	65.44	55.88
	72.54	68.04	80.13	67.24	66.22	70.54	65.08	62.62	54.87	58.09	69.38	66.76	57.98
	56.32	63.91	65.69	76.35	60.40	62.07	65.78	59.98	54.78	80.84	52.65	53.86	53.73
	62.64	58.24	60.38	65.27	63.44	56.65	50.65	65.02	66.92	65.55	58.49	63.06	56.46
	93.48	55.97	62.98	58.35									

Measurement data according to seed testing standards. Seed germination potential was calculated by counting the germination percentage of dried chili seeds on day 4. The seed germination potential of the high voltage electric field polarisation treatment test group was 12.50%. The germination potential of the untreated control was 3.41%; The number of germinated seeds of dried chili seeds was counted at day 14 to calculate the seed germination rate. Seed germination in the experimental group of high voltage electric field polarisation treatment was 84.09%. Seed germination in the untreated control was 63.64%; the Seed germination index of the experimental group treated with high-voltage electric field polarisation was 10.39. The seed germination index of the untreated control group with a high voltage electric field was 6.90; The average length of dried chili seedlings was measured by washing the seedling at day 14 to calculate the viability index of dried chili seeds. The mean length of dried chili pepper seedlings in the high voltage electric field polarisation treatment test group was 8.24 cm. The length of dried chili seedlings in the untreated control group was 6.37 cm. The seed germination index of the high voltage electric field polarisation treatment test group was 85.61. The germination index of the control group not treated with high voltage electric field was 43.95; Compared to controls not treated with high voltage electric fields. The germination potential of seeds in the high-voltage electric field polarisation test group increased by 9.09%, germination rate by 20.45%, germination index by 3.49, and vigor index by 41.66. All the seed viability indices of the high voltage electric field polarisation-treated test group were significantly improved compared to the non-high voltage electric field-treated control group. The results of the study can provide a basis for the selection of processes and parameters for subsequent high-voltage electric field polarisation treatment of crop seeds.

## Discussions

1. The results of a one-way test on the effect of different high-voltage electrostatic polarisation methods on seed viability showed that: Seed viability after negative output voltage polarisation was higher than after positive output voltage polarisation. Seed viability after positive output voltage polarisation was higher than after alternating positive–negative output voltage polarisation. Trace something to its source: The outside of the plant cell membrane has a high positive charge, and positive and negative charges are attracted to each other. Thus seed cell membranes are more sensitive to negative output voltage polarisation treatments. The selective semi-permeable function of the cell membrane is restored more rapidly when stimulated by an external negative high-voltage electric field, and the corresponding extravasation of mineral ions and organic matter from the seeds during soaking is reduced. Decreased content of conductive substances in seed leachate. Lower seed conductivity and higher seed viability. Under high-voltage electrostatic polarisation conditions, the selective semi-permeability of the cell membrane is altered, as can be verified in references^32–34^; However, the effects of different high-voltage electrostatic polarisation modes on the selective semi-permeability of cell membranes have rarely been reported. The mechanism by which the negative output voltage acts on the cell membrane and stimulates the restoration of the selective semi-permeability function of the cell membrane is not clear. We will examine this in more depth in subsequent studies.
2. Due to the potential difference between the inside and outside of the cell membrane. When the potential difference reaches a certain value can stimulate the cell membrane to restore its selective semi-permeability function. Altering the permeability of the cell membrane, regulating the bioelectrical properties of seed cells, and accomplishing intra- and extracellular migration of dielectrics. This can be verified in reference^29^. When the output voltage of the high-voltage electrostatic generator is small or the polarization time of the high-voltage electric field is short. The electric field was not sufficient to stimulate the cell membrane to fully restore its selective semi-permeable function, resulting in partial extravasation of mineral ions and organic matter from the seeds during soaking. Higher seed conductivity and lower seed viability. When the output voltage of the high-voltage electrostatic generator is large or the polarization time of the high-voltage electric field is long. Reversible perforations will be formed on the surface of the cell membrane, leading to partial extravasation of mineral ions and organic matter from the seed during soaking. Higher seed conductivity and lower seed viability. When the output voltage is 12 0.08 kV and the high voltage field polarisation time is 30.32 s. Electric fields stimulate the cell membrane to maximize the restoration of selective semi-permeable function and control intra- and extracellular electrolyte transport. Resulting in less mineral ion and organic matter extravasation from the seed during soaking. Lower seed conductivity and higher seed viability. This can be verified in references^32,35^, where the highest seed viability was observed when the output voltage was 12 kV; However, in references^36,37^ the highest seed viability was observed when the output voltage was 16 kV; This manuscript and references^32,35^ were studied for chili seeds and rice seeds, and the optimum output voltages were similar for seeds of similar size; References^36,37^ were studied on cotton seeds, which are larger and have a higher optimum output voltage than chili seeds and rice seeds compared to chili seeds and rice seeds. It can be seen that the optimal output voltage shows a positive correlation with the seed size. However, the migration mechanism of intra- and extracellular electrolytes under the modulating effect of an applied electric field is unclear. We will examine this in more depth in subsequent studies.
3. In this study, a fitted regression equation between seed conductivity and the output voltage of the high-voltage electrostatic generator and the polarization time of the high-voltage electric field was established. Rapid evaluation of the viability of dried chili seeds under conditions of high voltage field polarisation treatment of dried chili seeds. However, for other crop seeds, a rapid evaluation model for seed viability assessment has not been developed. We will construct a rapid evaluation model and method for multi-seed vigor in the course of subsequent research.

## Concludes

1. To study the effect of different high-voltage electric field polarisation process parameters on the viability of seeds of dried chili pepper production. In the present study, a high-voltage electrostatic polarisation system was constructed to carry out the effect of different high-voltage electric field polarisation treatment process parameters on the viability of seeds of dried chili pepper production. A two-factor, five-level central combination test was conducted using the output voltage of the high-voltage electrostatic generator the polarization time of the high-voltage electric field as the test factors, and the seed conductivity as the response indicator. The test results show that a high-voltage electrostatic generator output voltage of 12.08 kV and a high-voltage electric field polarisation time of 30.32 s are preferred parameter combinations. At this time the seed conductivity was a minimum of 159.87 uS/(cm g) and the seed viability was optimum.
2. In this study, we investigated the effect of different high-voltage electrostatic polarisation modes on the seed viability of dried chili peppers at a high-voltage electrostatic generator with an output voltage of 10 kV and a high-voltage electric field with a polarisation time of 15 s. The results of this study are summarised in the following table. The test results show that: Seed viability after negative output voltage polarisation was higher than after positive output voltage polarisation. Seed viability after positive output voltage polarisation was higher than after alternating positive–negative output voltage polarisation. Seed viability was higher than the control after different polarisation modes.
3. Under the conditions of negative voltage polarization, high voltage electrostatic generator output voltage of 12.08 kV and high voltage electric field polarisation time of 30 s. Compared to controls not treated with high-voltage electric fields. The germination potential of seeds in the high-voltage electric field polarisation test group increased by 9.09%, germination rate by 20.45%, germination index by 3.49, and vigor index by 41.66. Seed viability indices were significantly higher in the high voltage field polarisation test group compared to the non-high voltage field treated control group. The results of the study can provide a basis for the selection of processes and parameters for subsequent high-voltage electric field polarisation treatment of crop seeds.

## Data Availability

All data generated or analysed during this study are included in this published article (and its Supplementary Information files).
